# Machine learning based IoT system for secure traffic management and accident detection in smart cities

**DOI:** 10.7717/peerj-cs.1259

**Published:** 2023-03-08

**Authors:** Saravana Balaji Balasubramanian, Prasanalakshmi Balaji, Asmaa Munshi, Wafa Almukadi, T. N. Prabhu, Venkatachalam K, Mohamed Abouhawwash

**Affiliations:** 1 Department of Information Technology, Lebanese French University, Erbil, Iraq; 2 College of Computer Science, King Khalid University, Abha, Saudi Arabia; 3 Cybersecurity Department, University of Jeddah, Jeddah, Saudi Arabia; 4 Department of Software Engineering, University of Jeddah, Jeddah, Saudi Arabia; 5 Department of Information Technology, Sri Ramakrishna Engineering College, Coimbatore, Tamilnadu, India; 6 Department of Applied Cybernetics, Faculty of Science, University of Hradec Králové, Hradec Kralove, Czech Republic; 7 Department of Mathematics, Mansoura University, Mansoura, Egypt; 8 Department of Computational Mathematics, Michigan State University, East Lansing, MI, United States

**Keywords:** Smart cities, Internet of Things, Deep learning, Traffic management system, Accident detection, Secure early traffic-related EveNt detection, Adaptive traffic management system

## Abstract

In smart cities, the fast increase in automobiles has caused congestion, pollution, and disruptions in the transportation of commodities. Each year, there are more fatalities and cases of permanent impairment due to everyday road accidents. To control traffic congestion, provide secure data transmission also detecting accidents the IoT-based Traffic Management System is used. To identify, gather, and send data, autonomous cars, and intelligent gadgets are equipped with an IoT-based ITM system with a group of sensors. The transport system is being improved *via* machine learning. In this work, an Adaptive Traffic Management system (ATM) with an accident alert sound system (AALS) is used for managing traffic congestion and detecting the accident. For secure traffic data transmission Secure Early Traffic-Related EveNt Detection (SEE-TREND) is used. The design makes use of several scenarios to address every potential problem with the transportation system. The suggested ATM model continuously modifies the timing of traffic signals based on the volume of traffic and anticipated movements from neighboring junctions. By progressively allowing cars to pass green lights, it considerably reduces traveling time. It also relieves traffic congestion by creating a seamless transition. The results of the trial show that the suggested ATM system fared noticeably better than the traditional traffic-management method and will be a leader in transportation planning for smart-city-based transportation systems. The suggested ATM-ALTREND solution provides secure traffic data transmission that decreases traffic jams and vehicle wait times, lowers accident rates, and enhances the entire travel experience.

## Introduction

There are 1.35 million mortality cases reported each year globally, accounting for 2.2 percent of the global population (Lilhore et al., 2022). Each year, more deaths and instances of permanent impairment are due to everyday traffic accidents. The primary causes of traffic accidents are speeding, careless driving, driver exhaustion, stray animals on the roadways, and inadequate infrastructure. Due to the emergency medical services’ slow reaction, most fatalities and impairments in these incidents occur. The period immediately following a traumatic injury is referred to as the “golden hour,” during which rendering immediate, lifesaving surgery and medical care raise the likelihood of human survival by a mean of one-third (Olariu & Popescu, 2021).

The IoT for Information Technology Management (ITM) is now directly connected to many objects, including automated vehicles, cooperative transportation systems, and intelligent roadways, which improves data transmission and creates diverse connectivity and low-bandwidth gadgets in high-capacity locations all over the world. India is an emerging nation, and according to NSO research (Mateen et al., 2022), its GDP will decline by 7.7.

A frequently employed method of resolving traffic management difficulties is an ITM system. ITM systems can improve logistics and passenger transportation by reducing traffic congestion. The innovations used to raise people’s standards of living will determine how smart and long-lasting the growth of IoT-based ITM Systems is. ITM initiatives must include smart-city governance, which develops planning techniques for improved laws. The social policy of innovative services is one of the critical tenets of the smart-city management system (Hina, Soukane & Ramdane-Cherif, 2022).

IoT refers to connecting physical objects to the internet to build intelligent networks and wireless transmission connections using cutting-edge technologies like ITM. A novel information exchange model that supports ITM is communication among IoT-based vehicles. IoT combines data collecting, sensor data processing, and computation to handle successfully and sustain traffic networks (Jelínek, Čejka & Šedivỳ, 2022). A timer for each phase in autonomous transportation includes a traffic light, though. Another technique for tracking cars uses electronic detectors. Road traffic exists despite the employment of computerized traffic-control sensors to manage it. An intelligent transportation system can resolve traffic jams and other challenges (Parihar, Dasari & Bhagwat, 2022).

An AALS system is suggested in this study to identify car accidents and notify nearby automobiles. Therefore, because the entire structure is situated on the other side of the road, no modifications to new or EVs are necessary, giving rise to the novel idea of smart roads. An intelligent road can detect any incident on the road. The AALS system uses the event-driven wireless sensor network (EDWSN) protocol (Olayode et al., 2022) to communicate among nodes inside the event of an incident. Usually, every node is separate from the others. To find event, every node functions independently. The AALS system recognizes an accident when it occurs on a portion of an SR using its sensing capabilities and delivers light and audible alerts.

Intelligent cities allow AI, DL, and IoT-based systems, including embedded linked devices, IoT sensors, intelligent traffic-control systems, intelligent streetlamps, and intelligent roadways, to collect and analyze data. These metropolitan regions use the information obtained to optimize their public utilities, infrastructure, and things that communicate with various platforms, including Industry 4.0, intelligent buildings, smart cars, brilliant agricultural production, and innovative healthcare systems. This article shows the design and implementation of an ATM based on DL and IoT.

The main contribution of the proposed method is given below:

- The proposed method uses an accident alert sound system (AALS) to detect accidents and alert vehicles in smart cities.
- Identifies current traffic circumstances and identifiable patterns in the flow of traffic, allowing SEE-TREND to forecast impending traffic events and notify relevant parties of their tendency to occur.
- One of its key advantages is the capability of the suggested ATM-ALTREND structure to interact with any adaptable approach without requiring changes to the traditional architecture.

The rest of our research article is written as follows: Section 2 discusses the related work on smart city transportation systems, accident detection, and machine learning methods. Section 3 shows the general working methodology of the proposed work. Section 4 evaluates the implementation and results of the proposed method. Section 5 concludes the work and discusses the result evaluation.

## Related works

The authors of Bhatia et al. (2022) described an intelligent transmission-control system using cloud viewpoint and ML techniques. The cloud picture API is used to identify the concentration and driving experience. Its following traffic intersection’s visuals are recorded and preserved in the cloud database. Additionally, the situation is transferred to the next traffic light. The previous traffic signal, which is now operating, will monitor how the next traffic light performs and then carry out the activity by the circumstances.

The researchers of Kaginalkar et al. (2021) showed that all these techniques could assist us in anticipating traffic behavior, automated traffic-signal administration, parking recognition, and detection of surrounding objects/vehicles, all of which can boost the safety and effectiveness of ITM. However, improving traffic control is still challenging (Silva, Andrade & Ferreira, 2020). Numerous studies focusing on IoT-based intelligent traffic-control systems have been carried out. Increased traffic monitoring systems have been shown by the writers of Gatto & Forster (2020), to alter bright metropolitan regions.

According to Cao & Wang (2020), the foundation of services and facilities for urban planning is automatic traffic detection. Intelligent connection sensor networks calculate traffic flow, foresee traffic snarls, and adaptable manage traffic flow. Once done correctly, this raises awareness to a point where resources and equipment may be used more efficiently. In Schneider, Lugner & Brandmeier (2019), road traffic, utilization, and average densities extensively used vehicle congestion assessments. The majority of this data was gathered from images and videos captured by machine vision software. For this specific situation, the authors of Sequeira et al. (2020) presented an IoT-enabled monitoring system to collect, run, and consolidate real-world traffic patterns.

The researchers suggested an IoT-enabled control system (Bugeja et al., 2020) to acquire, manage, and aggregate real-world traffic conditions. Their main objective was to improve the range of motion while disseminating important traffic information about traffic jams and unforeseen crashes *via* the highway signaling system. A framework was used by Javed, Zeadally & Hamida (2019) to investigate the usefulness of the traffic state. The testing results show slight inaccuracy in the forecast of highway occupancy and good vehicle detection and tracking precision.

The development of deep automated learning (DL)-based system (Tian et al., 2020) for accident detection using visual information. The method uses temporally ordered graphic elements to illustrate traffic crashes. In conclusion, the system design consists of a phase for extracting visual features and identifying transient patterns. During the learning phase, sensory and spatial characteristics are learned using convolution and recurrent layers. An accuracy of 98% was reached in detecting accidents in publicly available road accident records, suggesting a great capacity for detection regardless of the road surface.

The detection of objects requires an intelligent platform to detect them objects effectively in the transport system (Zhang et al., 2021). The trajectory design using multilayer spatial networks achieves optimal solutions (Jia et al., 2022). Image detection requires intelligent semantic technology to detect objects in the urban background perfectly (Zhou et al., 2021). Recently digital twin algorithms are widely used in VANET for secure energy optimization (Chen, 2022). Due to improper traffic, the management environment is highly polluted and brings harm to human life (Xiao et al., 2020). Routing and navigation are key requirements of smart vehicle technologies (Xiao et al., 2021; Sun et al., 2021). For guiding the path, the Saccades Recommendation is used by drivers in urban areas (Xu et al., 2022). Recent developments in technology help blind people or sight-impaired people in traffic analysis and the selection of the optimal path (Xu et al., 2021; Ren et al., 2022).

The train is the biggest network and the transport system needs the utmost intelligence to control the traffic or delay in travel (Yin et al., 2022; Xiao et al., 2022; Zhuang et al., 2022). The features extracted for the traffic system are processed to wavelet filters to remove unwanted noises in features (Liu et al., 2022b). Wireless data transmission or wide band coverage become a major issue in traffic analysis applications (Liu et al., 2022a; Feng et al., 2022). Location tracking for logistics applications uses supervised learning techniques for object detection (Dai et al., 2022; Xie & Sun, 2022). The wireless communication system plays a vital role in transferring data in a secure and quick manner (Yang et al., 2022). Path planning using graph networks (Xie et al., 2022) helps in tracing an optimal path from source to destination (Cao et al., 2022). A deep learning-based classification algorithm for classifying roadside data is discussed in the article (Shen et al., 2020; Zheng et al., 2022b). This research collects roadside data and performs analysis for identifying traffic (Zheng et al., 2022a; Ban et al., 2022). The detection of objects (Li et al., 2021) using transfer learning can achieve an accuracy rate of more than 95. The fusion of sensors helps to predict the objects or collision objects in a better way (Du et al., 2021).

The issue only affects car accidents; motorcycles, bikes, and walkers are not included. Table 1 shows the various methods used for transport systems.

**Table 1:** Existing work based on IoT and various transport methods.

Author	Essential method	Algorithms used	Traffic congestion	Advantages
Dass, Misra & Roy (2020)	Identifying traffic congestion	ML and IoT	True	Intelligent route transfer and automated traffic detection techniques. The accuracy of identifying congestion must be done in time.
Deng et al. (2020)	Collision avoidance	Internet of Things, Big Data	True	Create a transport strategy that avoids collisions security in the network is not ensured
Cheng et al. (2020)	Intelligent transport system	ML, IoT	True	Nothing collided an improvement in rout ability enhanced safety suggest improved ML techniques for ensuring safety
Jan, Min-Allah & Düştegör (2021)	Pollution and collisions in traffic control	IoT, Deep Learning and Neural Networks	True	Energy-saving collision control technique optimized strategy can be used to identify the final solution
Asha et al. (2022)	Efficient, smart transportation	Cloud computing, IoT	True	Intelligent route finding with no collisions. Cloud alone cannot ensure the security of big data and processing time is not discussed
Bugeja et al. (2020)	Intelligent transportation planning	ML, IoT	False	Design for a smart city and parking infrastructure. Computation complexity is high
Ota et al. (2017)	Smart transportation and air quality	IoT, Cloud Computing	True	Pollution prevention and congestion management. Accuracy needs to be improved

## Proposed methodology

**Figure 1:**
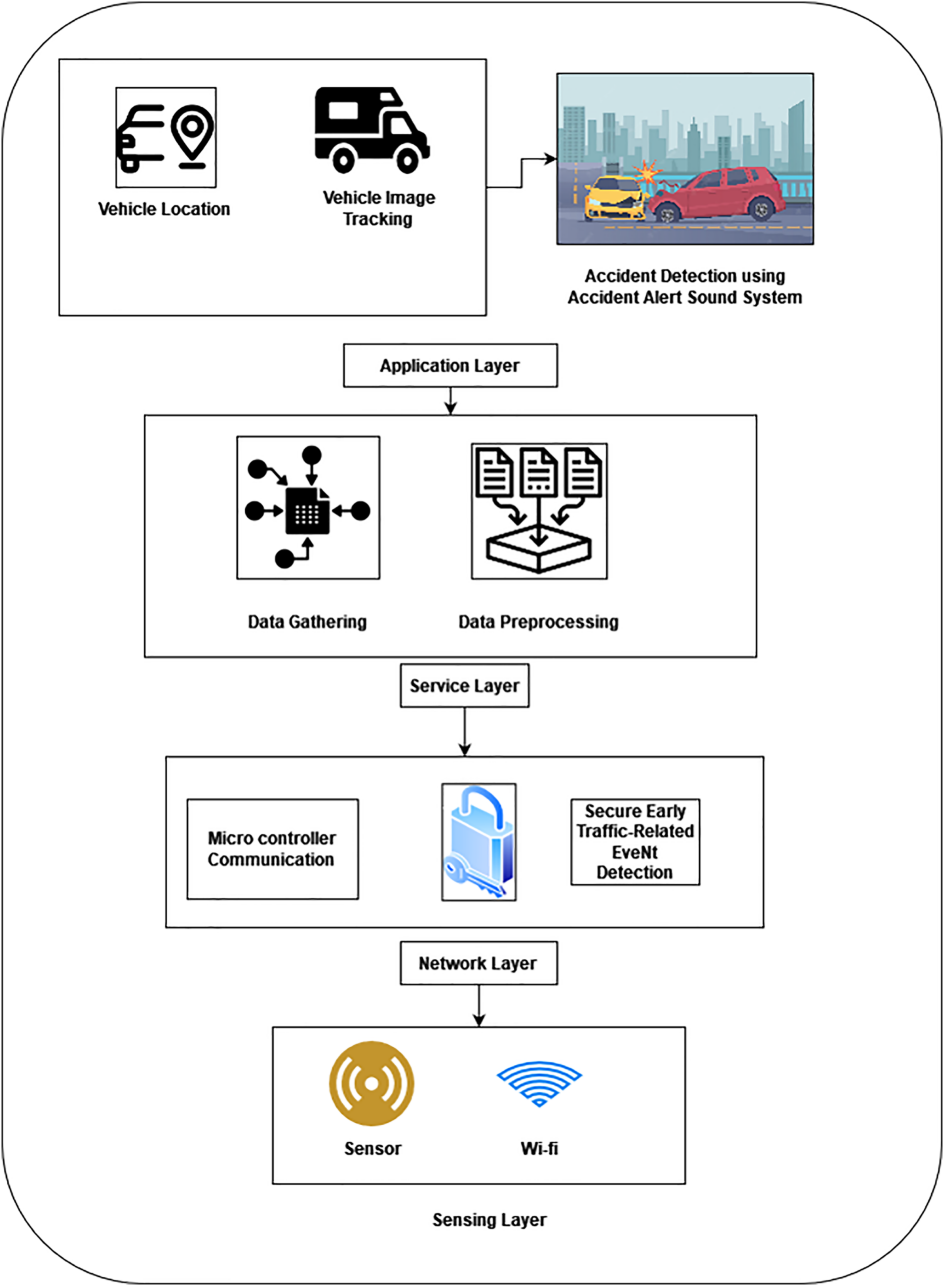
Architecture of proposed method.

The proposed method uses an Adaptive Traffic Management system (ATM) with an accident alert sound system (AALS) and Secure Early Traffic-Related EveNt Detection (SEE-TREND) for a secure and intelligent transport system. The proposed method consists of four layers for developing an innovative and safe transport system. Initially, the application layer monitors the vehicle’s location and image tracking, and then the accident alert sound system tracks the accident. The next layer is the service layer which gathers data, and then the collected data is pre-processed. The third layer is the network layer which is used for data communication. In this layer, Secure Early Traffic-Related EveNt Detection is used for transferring vehicle data securely. Finally, the sensing layer which collects the data with the help of sensors. Figure 1 shows the architecture of the proposed method.

### Application layer

In this layer, the vehicle location, vehicle image tracking, and then the attack detection phase is implemented.

#### Tracking the location of vehicles

**Figure 2:**
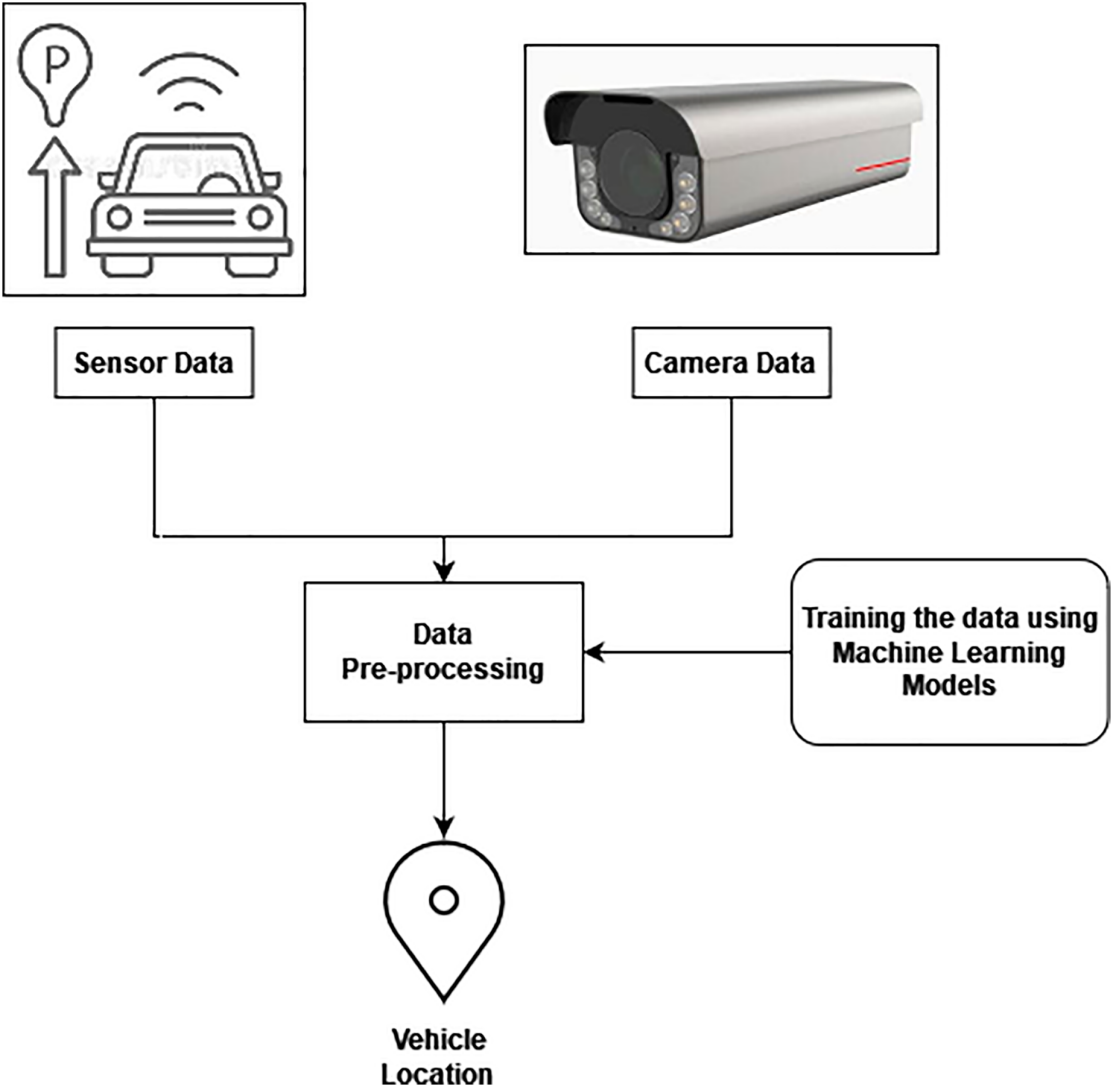
Tracking of vehicle’s location.

The suggested ATM-ALTREND system aids in selecting routes with greater accuracy. The lower limit precision level of the test is used to evaluate the quality of the model. However, let us assume the suggested model produces the lower bound with the appropriate level of precision. If so, it is evident that good, efficient pathways exist, and all other, less effective communication channels have been eliminated. However, there need to be more routes if the lower bound is higher than the anticipated precision rate. The set of essential ways for efficient vehicle positioning is expanded. The functional design of the proposed vehicle position monitoring system module is shown in Fig. 2. Information is gathered in the initial stage utilizing the sensors and photography equipment. Preparing data after sensors or cameras have captured it is an essential part of ITM. When preprocessing data, missing value estimate techniques are employed. The gathered data are processed using the processing method, and the dataset is then trained using the training method. Traffic information and the precise position of the vehicle are gathered.

#### Feature clustering

After tracking the locations of the vehicle, the feature clustering process is held. To avoid feature clustering, a graph is created. The nodes in the network (feature groups) reflect vehicle sightings, the edges represent connections between path clusters, and the nodes represent feature routes. For each trait and characteristic at a given time (TI $_i$):

- If the estimated total individual movement is sufficiently large, features discovered at a time interval (TI $_i$) for the frame (FR $_i$) are selected and tracked for a threshold number of frames. A Euclidean distance minimum connects almost all newly produced features that are extracted to the currently recorded characteristics.
- The higher and lower limit intervals are updated along with an approximation of the distance (Dist $_{i,j}$) between all currently monitored sets of connected functionality (LIf $_{i,j}$). The amount of the features separation threshold is represented by the Df $_{seg}$. The attributes of the connected automobiles are described in (1).

(1) $$\left[ {Max\;T{I_i}{d_{ij}}\left( {T{I_i}} \right) - Min\;T{I_i}{d_{ij}}\left( {T{I_i}} \right)} \right] \gt (D{f_{seg}})$$

- The related aspects of the graph are identified. Every combination of feature pathways that make up a related component, or vehicle observation, represents one. Let us assume that a component no longer has any functionality that is documented. The properties of the vehicle assumption (velocity vectors, centroid location, and vehicle type) are estimated after the characteristics are removed from the graphs.

### Accident detection using accident alert sound system

By using intelligent traffic control during Multiple Vehicle Collisions (MVCs), an ITM system can lower the likelihood of accidents and the number of unintentional fatalities (MVCs). To prevent MVCs, a system operating outside the car is required for accident detection and alarm generation for approaching vehicles. Here, a novel idea for creating intelligent highways is presented. Various types of sensors and actuators are fitted in smart roads (SRs) for the automated detection of accidents. Nodes in SRs (a separate system) are spaced roughly 50 m apart. You can use their previously saved placements since these nodes are attached to the road’s side of the road. To facilitate the quickest rescue effort and minimize damage, a node that notices an accident notifies its previously saved position to an EOC (Emergency Operation Centre). An AALS alert system that warns drivers of oncoming cars or accidents is the critical component of the SR.

The AALS is explicitly created for EVs and EVs to prevent MVCs. The majority of past research has attempted to develop a system that enables automobiles to feel one another and interact with one another *via* devices. In other words, they strive to equip every car. It is challenging to outfit all vehicles with the same communication and preventive system. A protection system installed at the manufacturer may be present in modern cars. The method employs a siren and a golden yellow blinking light to warn passing drivers of an accident.

Red and golden yellow light are seen from afar and in poor weather, claims one research of several colored lights. Since traffic lights already employ red lights, adding golden yellow light is a wise choice. A loud siren is another alternative method of warning motorists, allowing them to hear it and take action to prevent an MVC, particularly in BWC. These notifications are produced automatically. Both sides of the street will have the proposed system implemented.

**Figure 3:**
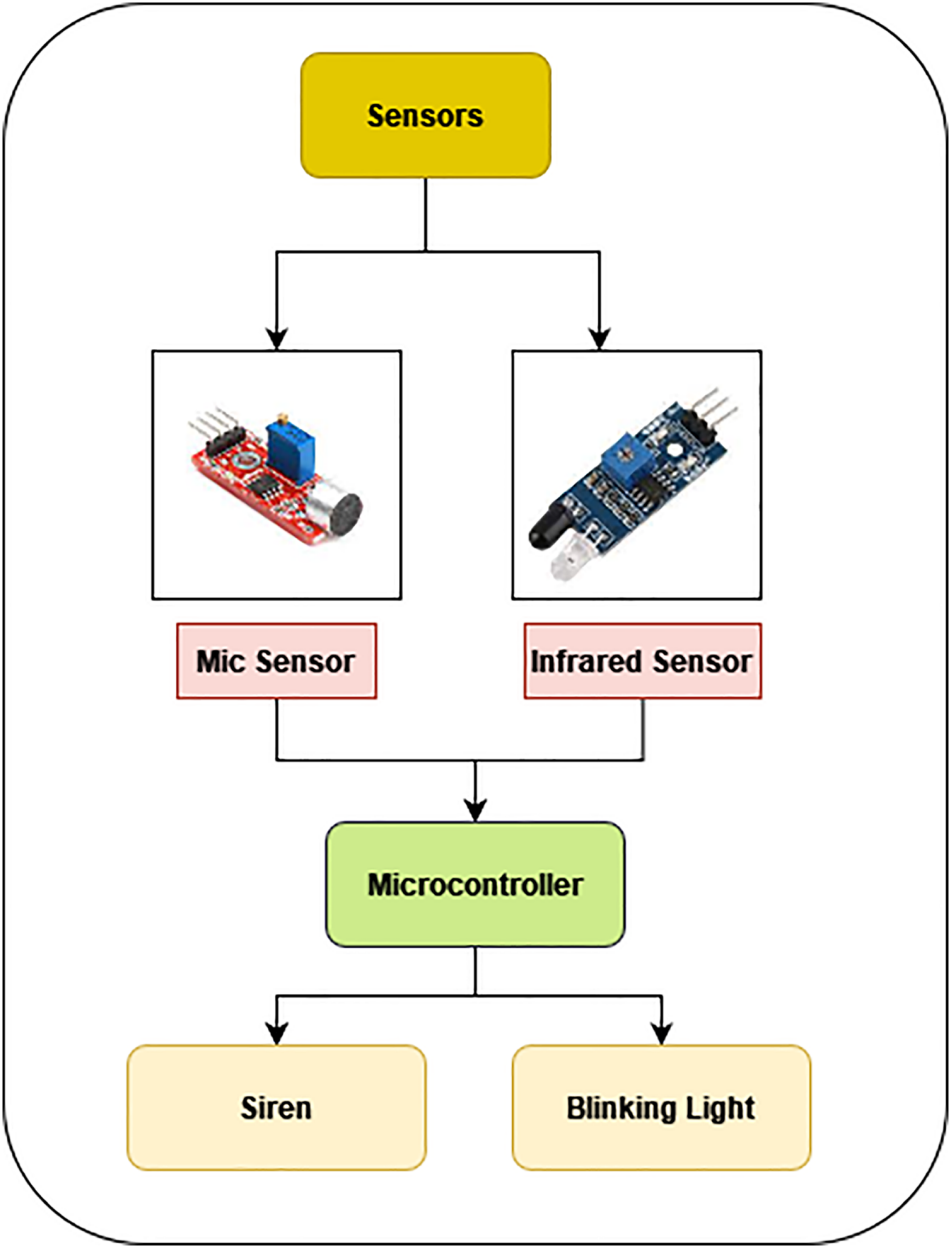
Block diagram of AASS module.

The primary purposes of the AALS technology are accident detection coming from external cars (*i.e*., from the side of the road) and accident notification to oncoming traffic *via* light and sound flashing. This only works whenever the reset button is hit or the AALS detects an accident. Certain things happen whenever an accident occurs. For instance, screeching noises are made when the brakes are applied quickly, and loud noises can be detected far away when a car collides with a different one. Glass shattering makes an audible noise; a burning car raises the atmosphere’s temperature and releases smog; if a vehicle immediately stops in the center of the road, it creates a hazard for other vehicles. A flawless accident detection system is available by considering all of these elements. The suggested method’s stages for network nodes are displayed. Figure 3 shows a basic block design of a node in the suggested AALS system.

The microcontroller receives input from the microphone, infrared, smoke, and HC-12 sensors. To find fires, a smoke sensor is employed. The microphones capture the audio and transmit it to the microcontroller that analyzes the levels with a predetermined threshold when it comes from a vehicle collision or hitting an item. If the level exceeds the threshold, an accident is declared. Therefore, the microcontroller dismisses the sound if it is not louder than the threshold. The IR sensors sense objects on the roadway concurrently in the interim. The microcontroller analyses the IR signal to see if it falls below the predetermined threshold and if more than 5 min have passed.

### Vehicle image processing in IMT

This technology uses pictures and electronic systems built into the roadways first to identify the automobiles. In addition to the traffic signal and sensors, the webcam will also be put in place. This will record visual data trends. Anomaly detection is the best choice to control the change in the status of the traffic light. A green signal over an empty roadway can decrease lost time and lessen traffic jams. Additionally, as it uses actual traffic-image data, it is more reliable at predicting the presence of vehicles. More significant than all other designs that rely on recognizing the vehicular surface material, it examines the usefulness and procedures.

### Vehicle communication

The sensors identify the car and its communication devices and continuously track its position in the traffic flow. Information is sent and shared amongst vehicles using IoT sensor devices in a manner that aids in reducing traffic and assuring travel safety. The platform is designed to alert users in advance of vehicular and dangerous driving situations, effectively handle accidents and fatalities, and address problems with data to be recognized by motorists on the road for safe traveling. Additionally, it is essential to communicate the information to the drivers by obtaining an astronomically large number of previously accurate statistics depending on the present traffic circumstances. In VANET, communication is crucial and is facilitated through the roadside unit (RSU).

### Secure traffic data using secure early traffic-related EveNt detection

Remember that SEE-TREND gathers traffic information from passing vehicles to become aware of conditions relating to traffic. Since the cars in localized cohorts experience the same traffic conditions, it should be no surprise that this information is strongly correlated. As a result, particularly in areas of heavy traffic, it is optional to gather data from every passing car; instead, we will focus on investigating the issue of probability collecting data.

Suppose that its Traffic Monitoring Unit (TMU) throws a biased coin that comes up tails with probability p and captures information from a particular moving car with probability *p* rather than gathering information from every passing vehicle. Assuming also that, for such application-dependent value >0, the data gathered from k cars results in the accurate detection of a traffic-related incident with probability $1 - {e^k}$. Let $A{E_n}$ become the instance when based on the aforementioned hypotheses, n passing cars are sufficient to accurately detect a traffic occurrence.

Let Hr serve as the random vector that determines the number of the n passing cars that have contributed traffic data. By training for the upcoming Hr, we now

(2) $$\eqalign{ {\rm{Pr}}\left[ {A{E_n}} \right]\; &= \sum\limits_{k = 0}^n {{\rm{Pr}}[A{E_n}|Hr = k]{\rm{Pr}}[Hr = k]} \cr& = \sum\limits_{k = 0}^n {\left( {\matrix{n \cr k \cr} } \right){p^k}{{(1 - p)}^{n - k}}(1 - {e^{ - ak}})} \cr & = \sum\limits_{k = 0}^n {\left( {\matrix{n \cr k \cr } }\right)} {p^k}{(1 - p)^{n - k}} - \sum\limits_{k = 0}^n {\left( {\matrix{ n \cr k \cr} } \right)} \;{(p{e^{ - \alpha }})^k}{(1 - p)^{n - k}} \cr & = 1 - {[1 - p(1 - {e^{ - \alpha }})]^n} \cr} $$

In addition, the vehicle arrival rate, which in turn is based on traffic flow, affects the value of *n*. A specific TMU can calculate the number n of entries per unit of time if it is informed of the frequency of traffic flow. This will then make it possible to calculate *p*. If the traffic flow intensity allows, the TMU can consciously choose not to gather information from a certain number of vehicles. This will decrease TMU power usage without affecting the time it takes to notice a problem connected to traffic.

#### SEE-TREND security solutions implementation

The SEE-TREND was created to permit the anonym collection of data while guarding against impersonation attempts. We now go over potential methods for ensuring security against DoS and confidentially threats.

Security from privacy attacks is included in SEE-TREND. Even from a position along the roadside, the attacker cannot understand the handshaking technique between a TMU and a moving car since it uses encrypted data. Establishing frequencies for communication between the TMU and the moving vehicles is an essential component of the handshake. By doing this, the opponent will be unable to understand the future data exchange.

Although these protection measures are currently in place, a variety of problems still exist and will be looked upon in this suggestion. Among the main challenges of SEE-TREND is minimizing the effects of DoS assaults, which can take many different forms. Two opponents, X and Y, acting simultaneously, each placed by the edge of the highway near neighboring TMUs A and B, try the following technique in one type of DoS attack: The keys for the new vehicle are all picked up by X and sent to Y *via* TMU A. Y will then communicate to B while posing as a legitimate vehicle.

Whenever the vehicles that provided the key try to connect TMU B, the request would be refused because the opponent has already utilized the one-time key, which TMU B will recognize as fake (as the time stamps do not match). This type of DoS attack could intentionally spread out all the traffic shown by TMU B if there are a lot of new cars, which would delay the duration it takes for SEE-TREND to identify an incident.

**Figure 4:**
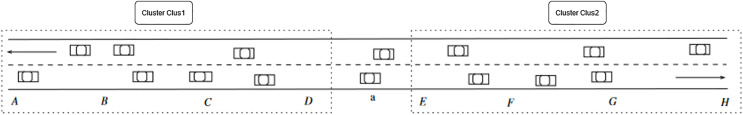
Clustering and fault tolerance in SEE-TREND are illustrated.

Fault tolerance is a significant concern in SEE-TREND. The fundamental question, in this case, is how SEE-functioning TRENDs may be maintained when a TMU is shut off due to a failure or energy outage. Assuming a vehicle moves among TMUs D and E on the two-lane road portion depicted in Fig. 4. These TMUs share the time-varying symmetric key $\rm \mu$ (D, E, t) that encrypts all messages sent between them. The data that D posted are lost if E is disabled because the subsequent active TMU, F, cannot decipher them. This effectively makes the vehicle “fresh,” which may negatively influence how quickly data is collected for SEE-TREND.

We next go over a potential method for implementing fault tolerance in SEE-TREND. As seen in Fig. 4, the goal is to create a straightforward clustering algorithm on the collection of TMUs by clustering together k, (k > 2), neighbouring TMUs. The clustering factor, or k, will be used from now on. Every TMU in this clustering method only joins one cluster, leading to fragmented clusters as a consequence.

According to the theory, communications can transmit successfully from end to end in accordance with the SEE-TREND semantics as soon as each cluster has an active TMU. The TMUs can be clustered either statically or dynamically. Each TMU is aware of the identity of the clusters that it participates in either scenario. Let’s examine a SEE-TREND made up of n successive TMUs with a clustering ratio of k, (1 $\le$ k $\lt$ n), to see how much fault tolerance our clustering strategy provides. The likelihood that, under incident X, SEE-TREND would failure to transmit messages end-to-end is a solid indicator of fault tolerance. To calculate this likelihood, we consider at the sequencing of n TMUs as a sequence of n characters A and D, where A stands for an active TMU and D for a disabled one. We then suppose that TMUs fail separately of one another with certain technology-dependent chance p.

(3) $$\sum\limits_{i = 1}^{{n \over k}} {\left( {\matrix{
   {{n \over k}}  \cr 
   i  \cr 

 } } \right)} {p^{ki\;}}{(1 - {p^k})^{{n \over k} - i}} = 1 - {(1 - {p^k})^{{n \over k}}} \approx 1 - {e^{ - {{n{p^k}} \over k}}}$$

After outlining SEE components, TREND’s following part goes into how the system achieves its stated goals.

### Mathematical model for ATM-ALTREND

The “platoon-based traffic flow” is the foundation of the suggested ATM-ALTREND model. A platoon is typically thought of as a group of cars traveling side by side, either voluntarily or involuntarily.

Each vehicle is predicted to receive an impartially required power V in the suggested ATM-ALTREND mathematical formula, and the traffic variance, FT (VE), is given. Automobiles travel continuously if they close the distance to the traffic in front of them. Once it has caught up, the object immediately slows down to match the speed of the moving object and follows it while maintaining steady progress.

**Figure 5:**
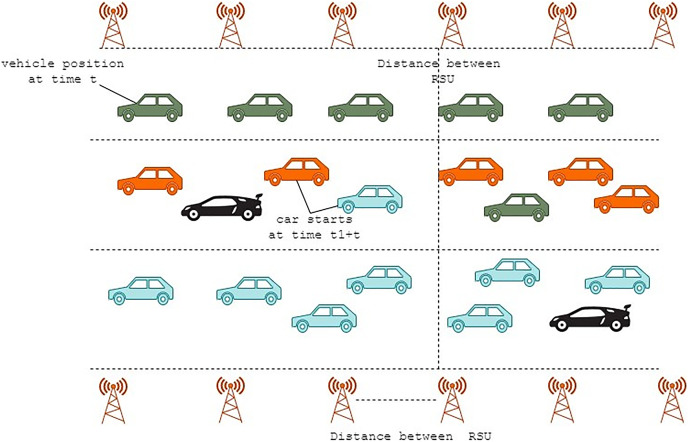
Proposed ITM system design.

The road traffic in terms of available capacity *vs*. time is depicted in Fig. 5. The road lane’s length is denoted by the letter LE. TV $^{In}$ denotes the time of entry, TV $_{out}$ denotes the time of exit for the vehicle, VE $_i$, and (TVh $_i$) denotes the time of headway for the vehicle, VE $_i$ indicated in Eq. (4).

(4) $$T{V^{out}} = \left\{ {\matrix{ {T{V^{ou{t_i}}}\;(for\;i = 0)} \cr {Max(T{V^{ou{t_i}}},\;T{V^{ou{t_{i - 1}}}} + TV{h_i}(for\;i = 1,2, \ldots ,n)} \cr } } \right.$$

In Eq. (5) it can be used to compute the time-out TV^out^. There are no cars near the boundary configuration when i = 0.

(5) $$T{V^{out}} = \{ TV_i^{in} + (L/V{E_i})\}$$

Let PV (EV $_s$) represent the probabilistic likelihood of an N-car platoon attempting to produce an EV event. Therefore, EV is the occurrence, and PV (N|EV $_s$) is the probabilistic likelihood that a platoon of N cars will cause event EVs to occur. At the conclusion of the evaluation phases, the likelihood $P{V^{PrimaryVE}}$ (N) that a particular new car will be the lead vehicle in a platoon of n cars given in Eq. (6).

(6) $$P{V^{PrimaryVE}}\left( N \right) = li{m_{0 - \infty }}PV\left( {EVs} \right)*PV\left( {N|EVs} \right)*{F_{VE}}(PV)dv$$

## Machine learning models for atm-altrend

Next the machine learning model is used for training and testing the traffic data. The significant infrastructure required for the development of the smart city, intelligent transport system is a hotly debated field of study (Ota et al., 2017; Datta & Sharma, 2017; Simaiya et al., 2020). Additionally, it employs IoT and ML technology to resolve disputes in a positive manner (Fig. 5). The dataset contains all relevant information about the traffic environment. It contains information on the car, the road, and the traffic. The following steps are involved in this process.

Step 1: The dataset contains vehicle data, road and traffic length and accident locations are collected initially.
Step 2: The collected dataset is pre-processed and then the pre-processed data is given into training.
Step 3: The trained dataset is given into Machine learning method. Here machine learning based DBSCAN clustering method is used.
Step 4: Next the trained model and testing dataset is evaluated. Finally the desired outcome is produced.

## Result analysis

A MATLAB simulator was used to construct the suggested ATM-ALTREND system. The suggested system makes use of the three main components (vehicles, infrastructures, and occurrences) listed in Table 2.

**Table 2:** Entities used for proposed transport system.

Entities	Sub-unit	Characteristics	Features
Vehicles	Two wheelers, three wheelers and four wheelers Vehicle Control Unit (VCU) Road Unit (RU)	ID of the vehicle, efficiency, type of vehicle and lane automated and manually ID of the lane, name of the lane, length of the lane, one way or two way	Indentifies the vehicles checks the control type of the vehicle describes the road unit
Infrastructure	Traffic light, control unit, street light unit	Installation status, ID of delay duration, speed of the vehicle	Describes the street light unit identifies the V2V communication
Events	Vehicle to vehicle communication Vehicle to Infrastructure communication	Signboard, traffic light, speed indicator	Identifies the V2I communication

Different traffic situations for linked autonomous vehicles (LAVs) were developed in order to verify the effectiveness and reliability of the suggested ATM system. (a) Only when connected to LAVs; (b) where few non-LAVs are present; (c) where both LAVs and non-LAVs are traveling. Figure 5 depicts the suggested ATM-ALTREND model’s design methodology for road congested roads with all of moving cars facing ahead.

The proposed method evaluates three scenarios such as only with LAVs, where only with non-LAVs, and where LAVs and non-linked both types of vehicles are moving.

*1. Only with LAVs:*

It is the first situation that solely takes LAVs into account. In this case, the traffic is primarily divided into two groups by the intelligent traffic-management systems. The control segment (CS) is the first, and merging segmentation is the second (MS). Control unit (CU), a component of the CS, aids in communication with LAVs (Lilhore et al., 2022).

*2. Where only with non-LAVs*

Evaluations are required to confirm the viability of suggested ATM-ALTREND techniques. In order to enable users to evaluate multiple viewpoints, a traffic virtual environment system must be simple to adjust to different traffic scenarios. On the basis of vehicular modeling, a baseline sequence of occurrences is created and assessed, with just fixed-cycle traffic lights monitoring non-LAVs.

*3. In which both kinds of vehicles—LAVs and non-linked—are traveling*

The mixed-traffic scenario, in which LAVs and non-LAVs use the same roads, should be seen as a major obstacle to the widespread adoption of automated cars. The suggested course of action in this circumstance is tested using system model control approaches.

In order to execute the planned ATM-ALTREND system, IoT sensors will gather real-time data on vehicular traffic and driving conditions and store it in a data center. Big data processing methods will be used to analyze the real-time data in the following phase. The final stage will rely on machine learning techniques to analyze the data. We presume that the wireless transmission component that communicates with the RSU in the suggested ATM-ALTREND system is fully installed in motor vehicles. Situated mostly on the highway, (Fig. 5) is designed to transmit substantial traffic information with other moving vehicles.

### Simulation environment

The simulated results show that the suggested ATM-ALTREND system works better in terms of the packet delivery ratio, bandwidth, and time delay than the traditional traffic-management system (Lilhore et al., 2022). This functions best when latency and traffic conditions (as in scenarios 1 to 3) change. The graphs below show the many factors and their importance in relation to specific system designs for communicating with packets of data. A 5,000 m road, 100 vehicles, and four different traveling directions (Channels 1: East to West, 2: North to West, 3: North to East, and 4: East to North) were used in the test, which produced the findings.

Traffic moves in both outflow and inflow directions in every simulation. The random model is the type of model. The likelihood of inbound and outbound traffic is 0.52% and 0.43%, respectively. Three separate scenarios’ simulation results were computed.

### Scenario 1

**Figure 6:**
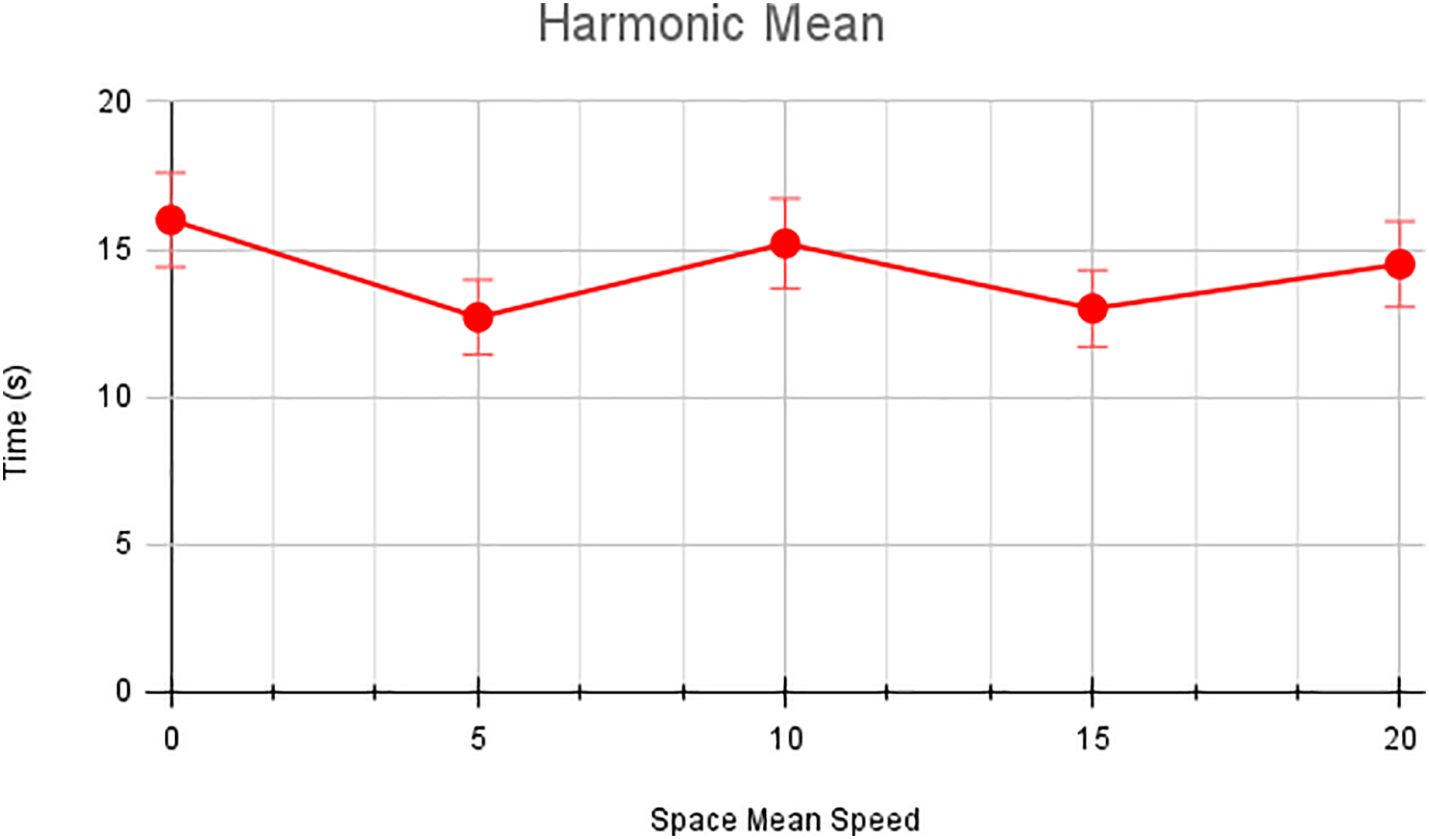
Harmonic speed of scenario 1.

**Figure 7:**
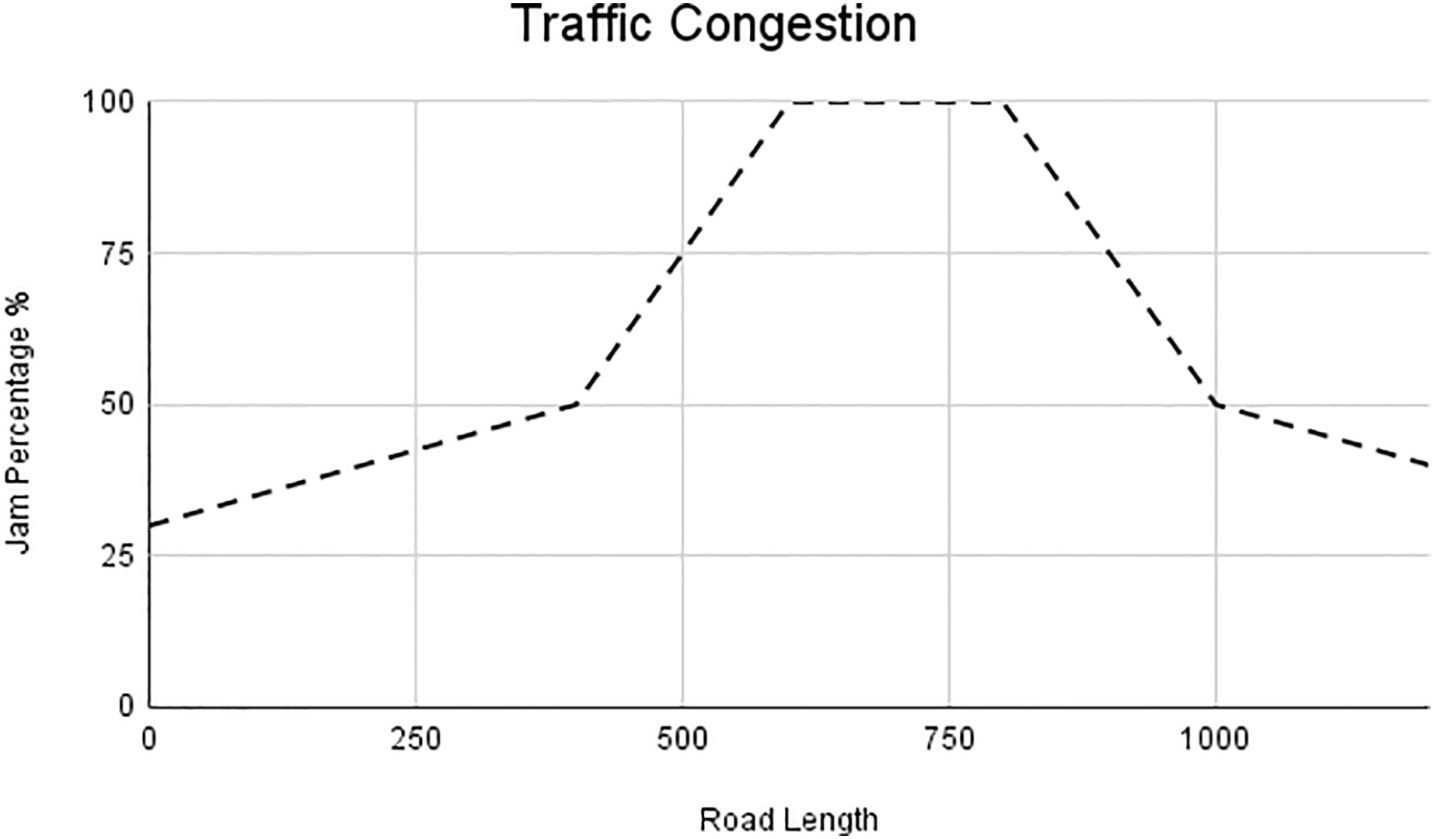
Traffic congestion of scenario 1.

**Figure 8:**
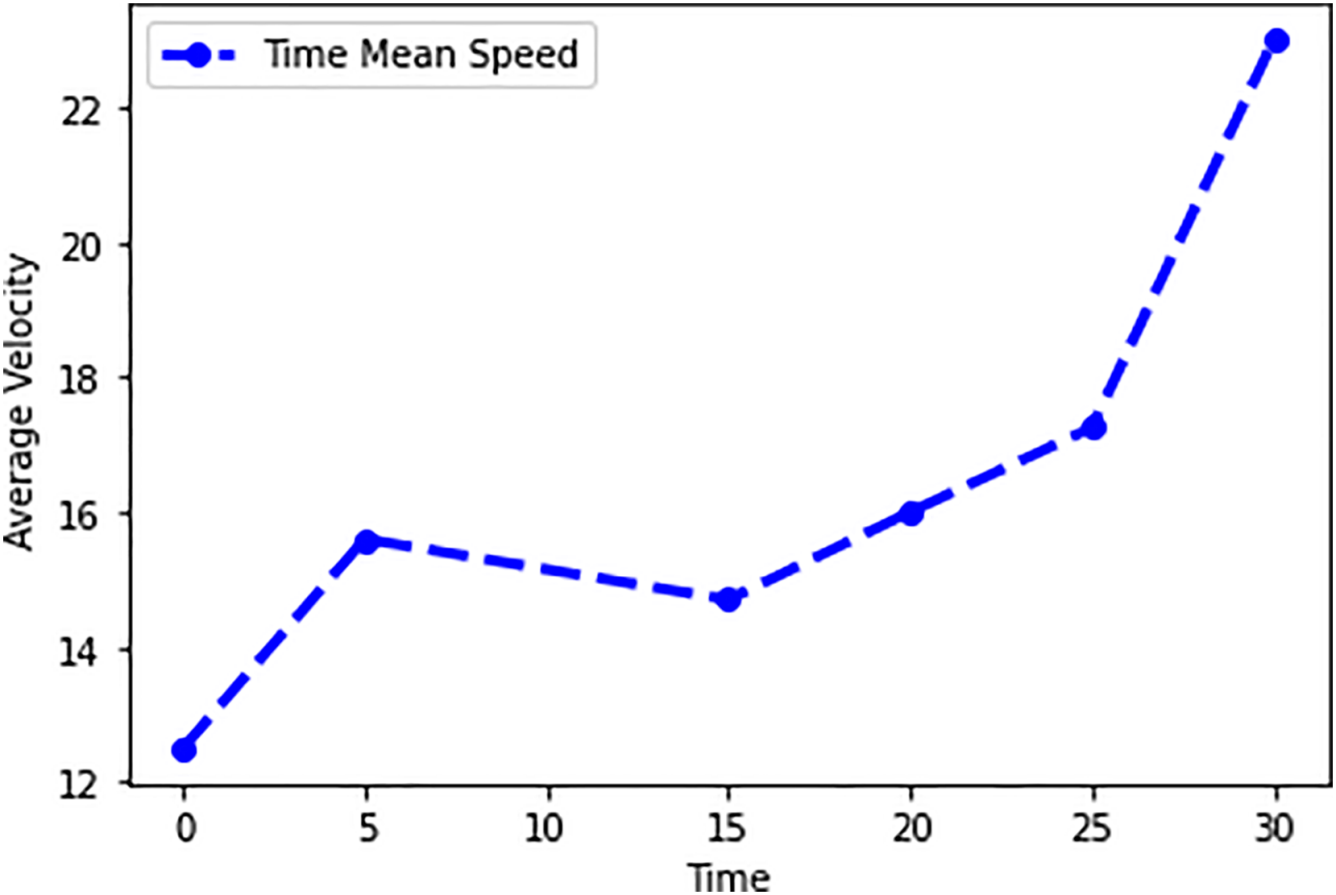
Time mean speed of scenario 1.

**Figure 9:**
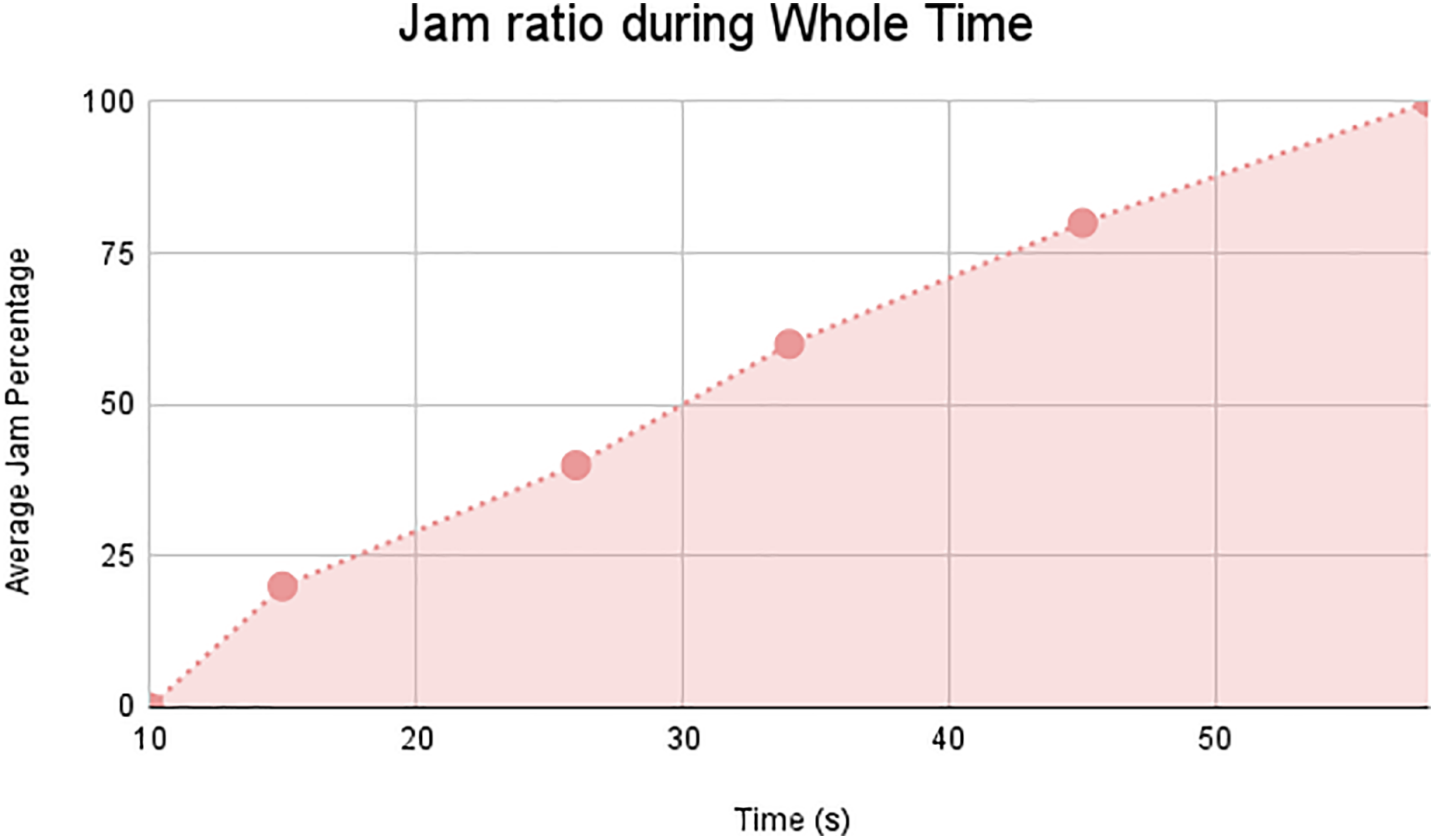
Jam ratio during whole time.

In this scenario, it is only with Linked Automated Vehicles (LAV). The experiment evaluates the traffic congestion ratio, time means speed, harmonic mean and jam ratio during whole time. From Figs. 6–9 shows the results of scenario 1. The proposed method has better performance in experimental results. It reduces the traffic congestion, jam ratio during whole time. Also it reduces the time mean speed and harmonic mean.

### Scenario 2

**Figure 10:**
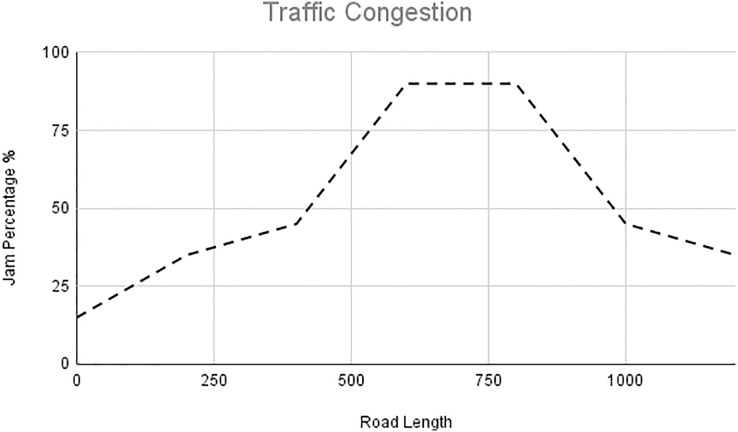
Traffic congestion of scenario 2.

**Figure 11:**
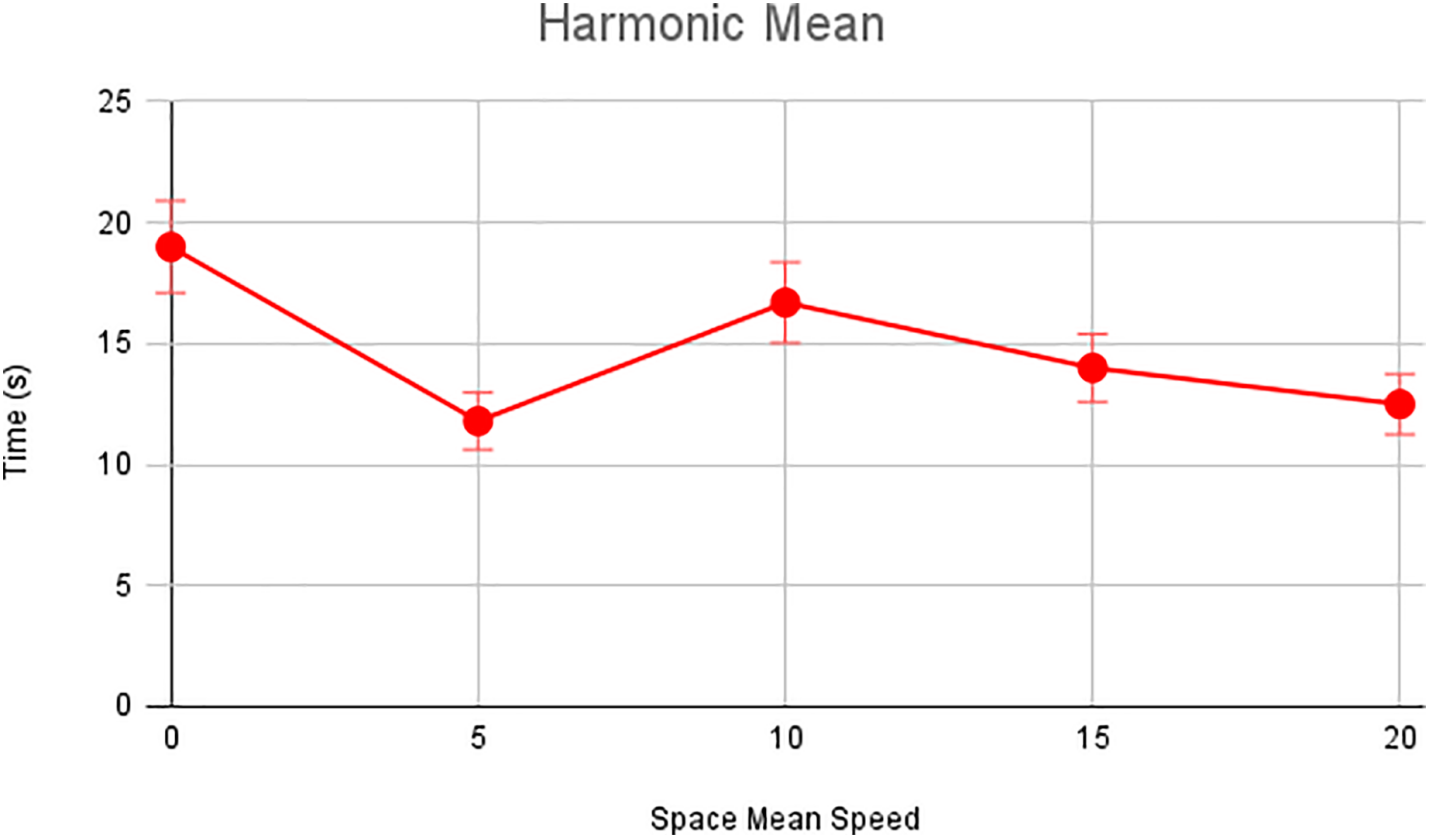
Harmonic mean of scenario 2.

**Figure 12:**
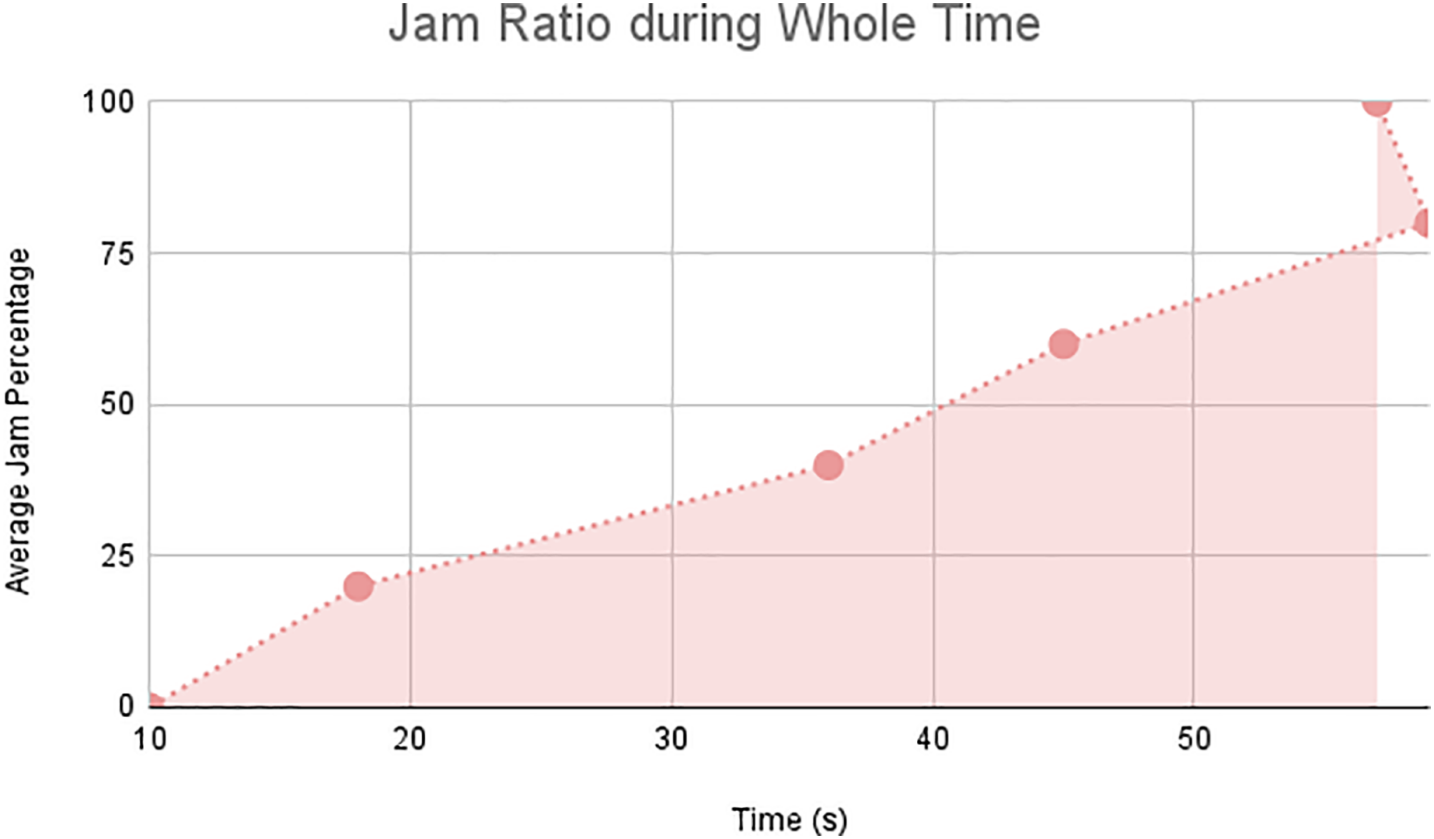
Jam ratio during whole time of scenario 2.

**Figure 13:**
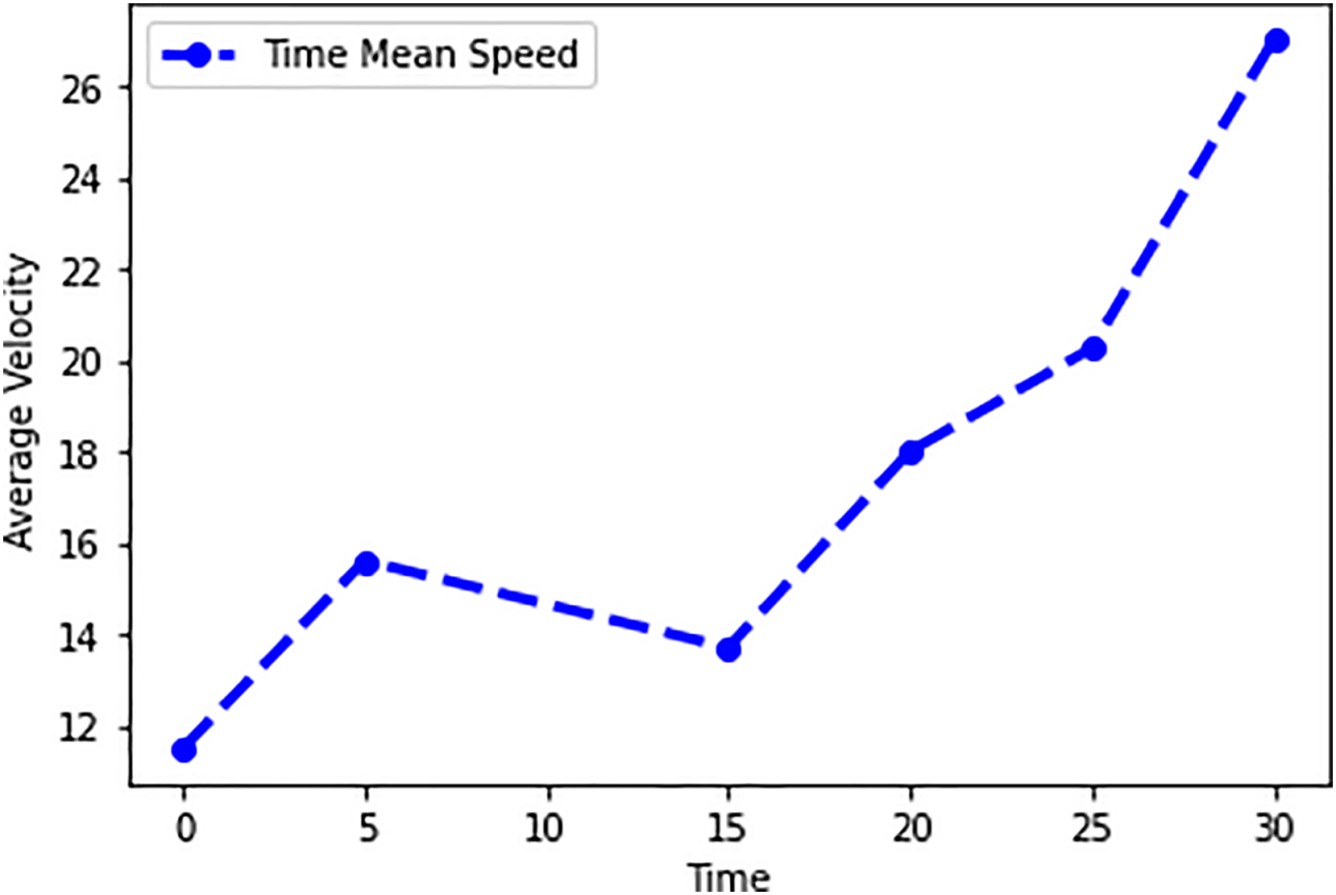
Time mean speed of scenario 2.

In this scenario it belongs to non-LAVs, the experiments were carried out by using the following metrics such as traffic congestion ratio, time mean speed, harmonic mean, and jam ratio during the whole time. Figures 10–13 shows the experimental results of scenario 2. The proposed method achieves better performance in all the parameters used for the transport system.

### Scenario 3

**Figure 14:**
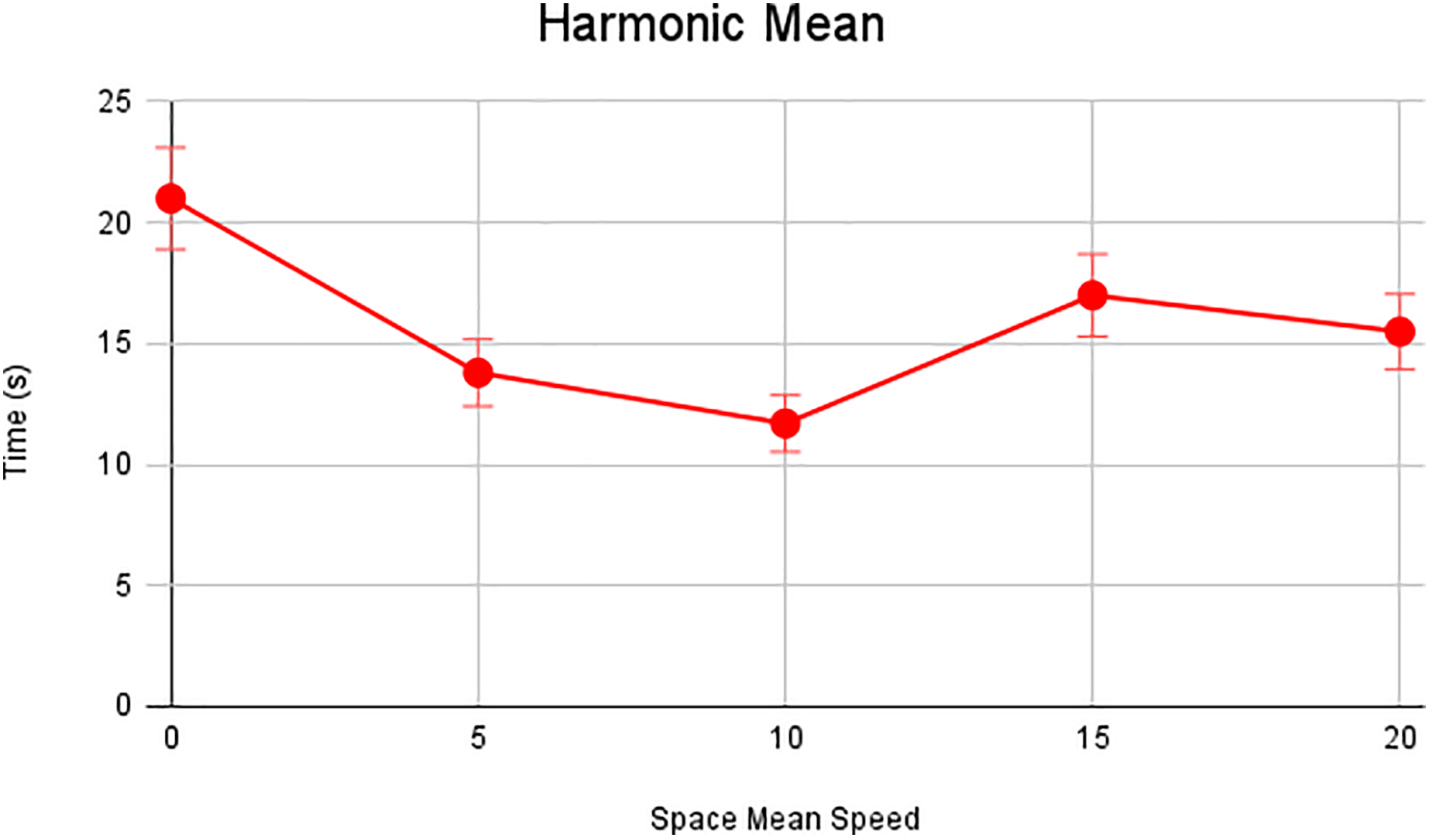
Harmonic mean of scenario.

**Figure 15:**
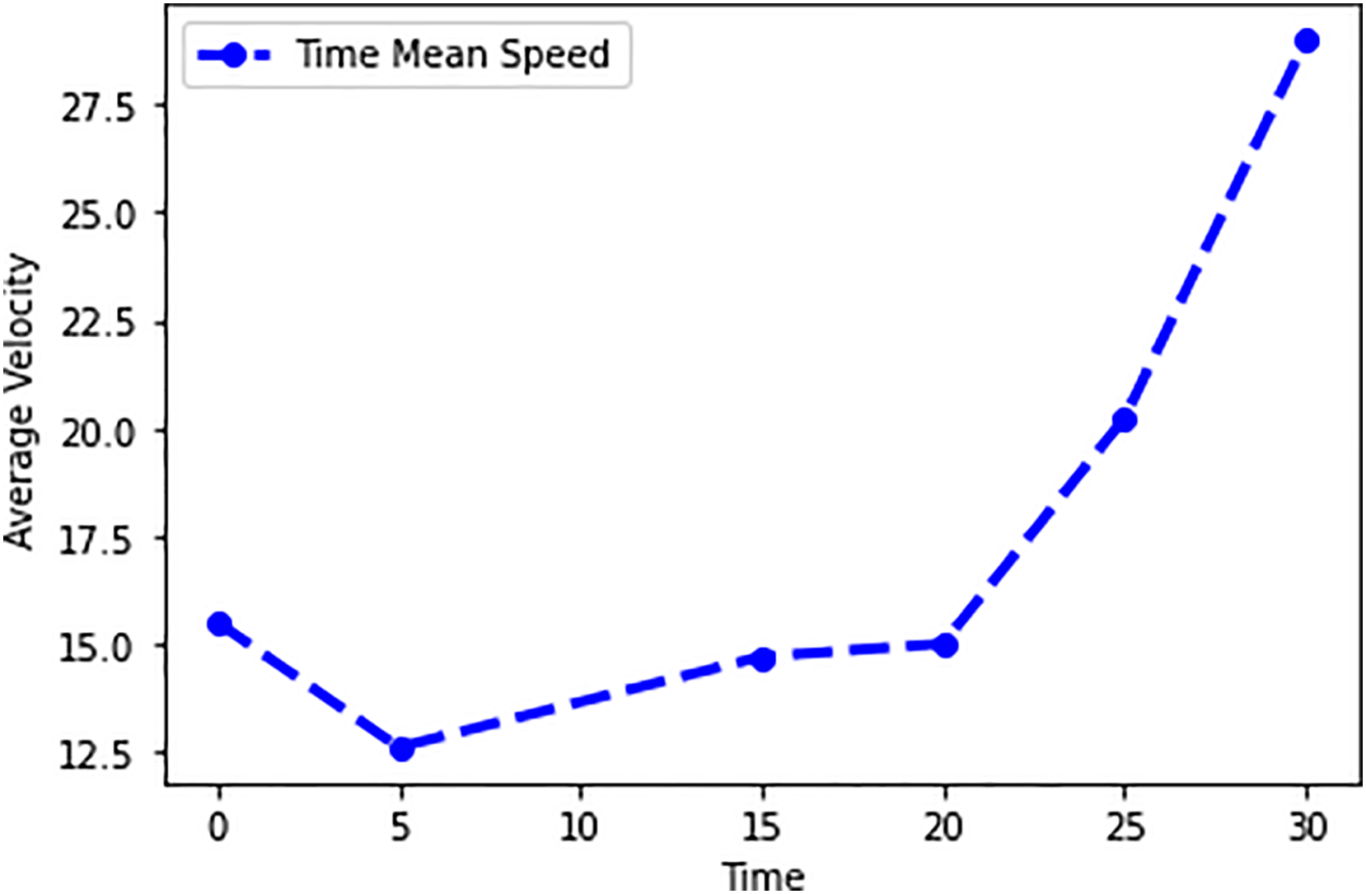
Time mean speed of scenario 3.

**Figure 16:**
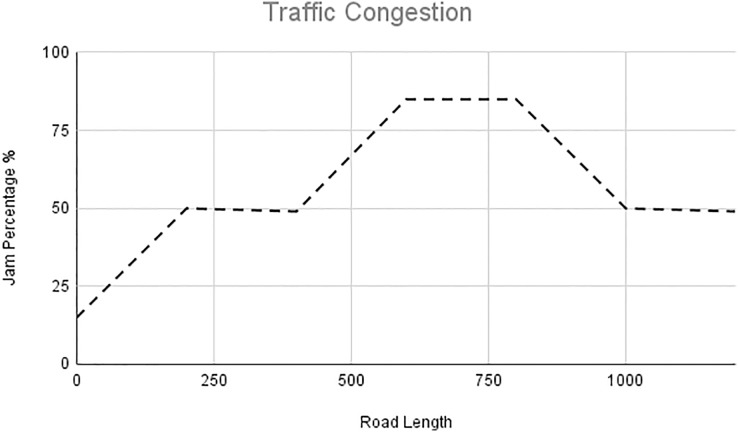
Traffic congestion of scenario.

**Figure 17:**
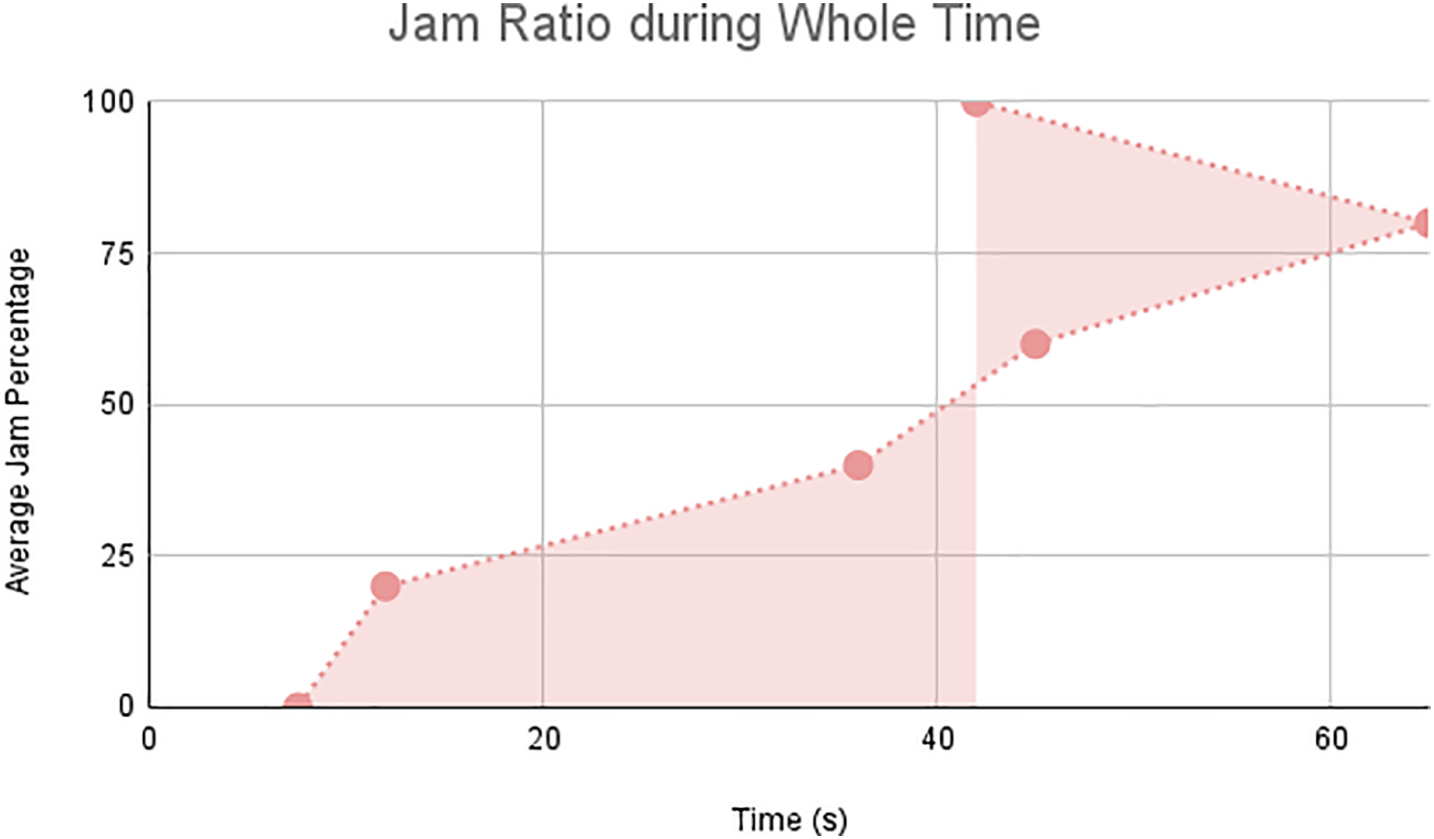
Jam ratio during whole time of scenario 3.

In this scenario it belongs to both kinds of vehicles—LAVs and non-linked—are traveling, the experiments were carried out by using the following metrics such as traffic congestion ratio, time mean speed, harmonic mean, and jam ratio during whole time. Figures 14–17 shows the experimental results of scenario 3. The proposed method achieves better performance in all the parameters used for transport system.

### Confusion matrix for clustering DBSCAN and machine learning method

**Figure 18:**
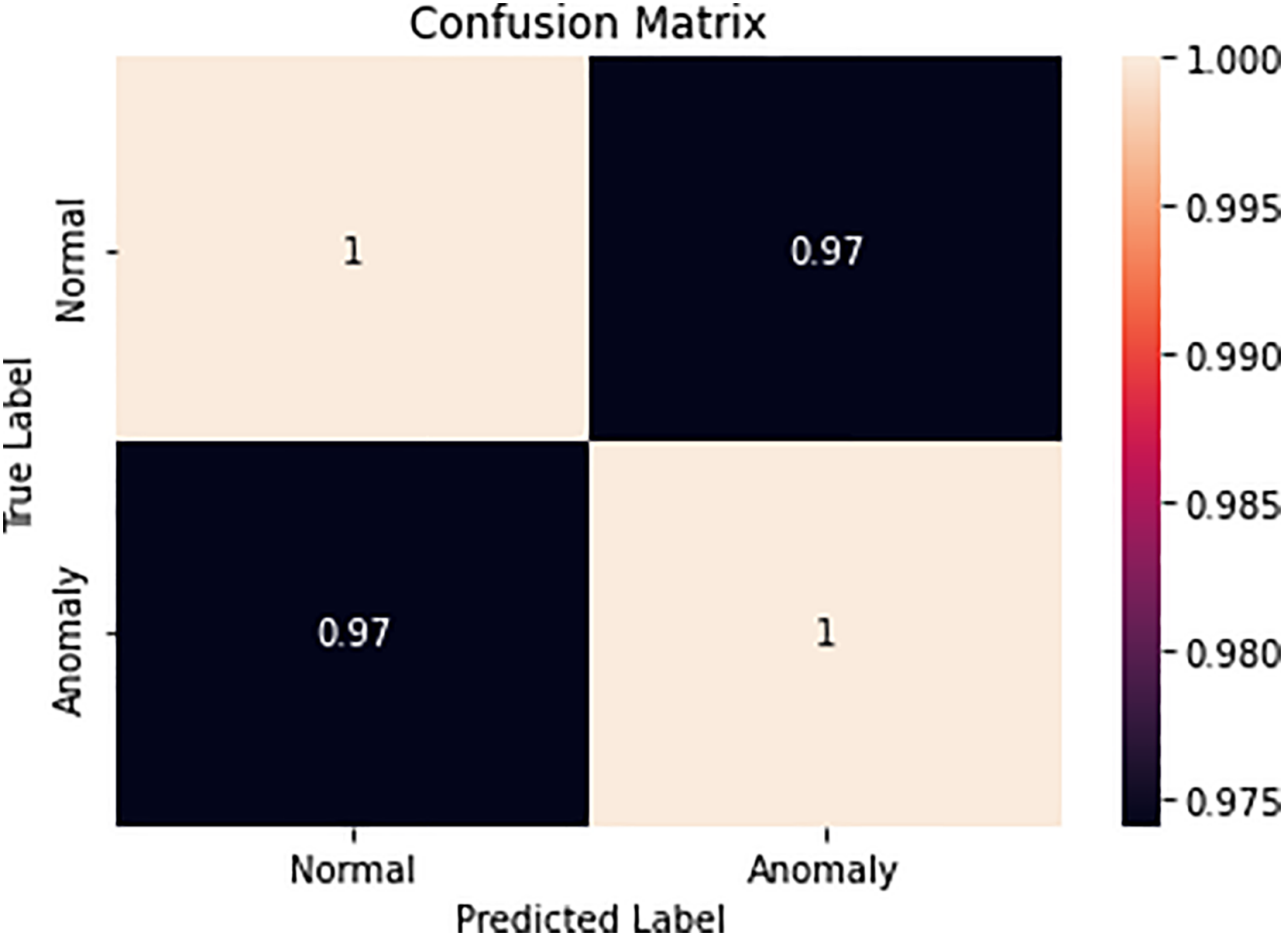
Confusion matrix.

A traffic simulator without collisions is the MATLAB simulator. A DBSCAN technique based on machine learning will be used to find the accident in the following stage. The clustering results of DBSCAN and ML techniques for accident avoidance are shown in Table. A vehicle is under the pressure to stop in a specified spot. Complete stops can also be seen as important accidents on a particular stretch of the road. Transportation systems can be complicated by vehicles operating alone, by drivers who are also passengers, or both. Potential crashes can be avoided by recognizing such situations and activating approaching vehicles. Each vehicle in the simulation must come to a complete stop at a road view every 95 s. In Fig. 18 the confusion matrix of the clustering method is evaluated. The clustering method uses two types of clusters normal and anomaly. The proposed method ATM-ALTREND detects the normal and anomaly cluster type among the vehicle count.

## Conclusion

In this work, an Adaptive Traffic Management system (ATM) with an accident alert sound system (AALS) is used to manage traffic congestion and detects an accident. The intelligent transport system alerts vehicles during accidents and minimizes collisions during traffic. Secure Early Traffic-Related EveNt Detection (SEE-TREND) is used for secure traffic data transmission. The ATM-ALTREND model that has been developed provides.

- On-site auto operations.
- Smart parking.
- The application of traffic-management strategies for the creation of an intelligent transportation system.

The plan aids in tracking vehicle travel, allowing for the analysis of traffic in a particular area. The SEE-TREND minimizes intrusion and secures the traffic data during data transmission. In this work, three scenarios are when connected to LAVs, where few non-LAVs are present, and where both LAVs and Non-LAVs are traveling. In these three scenarios, the proposed ATM-ALTREND outperforms better than the existing work. Vehicle traffic management systems have been increasingly interested in automatic accident detection. Keeping an eye on a collision will help us prevent future occurrences that might be similar, and it will make it easier for security services to reopen the road segment to various vehicles. With the use of vehicle locations and average speeds, we were able to demonstrate that traffic activity could be assessed convincingly. Drivers closest to the accident scene may also view unusual roadway incidents as a potential threat. It was discovered that the suggested ATM-ALTREND system outperformed the currently used traditional processes.

## Supplemental Information

10.7717/peerj-cs.1259/supp-1 Supplemental Information 1 Code.

## Additional Information and Declarations

### Data Availability

The following information was supplied regarding data availability: The Road Safety Data is available at: https://www.data.gov.uk/dataset/cb7ae6f0-4be6-4935-9277-47e5ce24a11f/road-safety-data.
